# Carbolic Acid Spray in Coughs

**Published:** 1878-07

**Authors:** 


### EXCERPTA.

Carbolic Acid Spray in Coughs.—A correspondent, who is a druggist in this city, sends us the following communication on this subject:

More than a year ago I read in the *Journal of Chemistry* a statement that carbolic acid in the form of spray benefitted a cough. Having a severe cough at that time, I used the acid as directed, of a strength of about two per cent., with an atomizer, but finally tried five per cent., or the saturated solution. I took no medicine, and the cough went away in a few days. Now, from my first recollection I have had severe coughs, and have always had bronchitis, for which I have taken much medicine; but since using the carbolic spray I have had no cough for a year. If I feel any of the symptoms which precede a cough or a cold, a few inhalations remove all the disagreeable feelings, and prevent a cough. Inhalation through the nostrils stops sneezing and the flow of mucus. I have recommended it to many others, all of whom were benefitted, and cured if they continued to inhale the spray.

I have called the attention of many physicians to the value of carbolic acid in coughs, asthma, and chronic catarrh, and to the fact that the saturated solution (five per cent.) could be used with safety, and would in most cases be more beneficial than a weaker solution. They have answered that they would not give the acid of that strength under any consideration. But I have often used it of that strength, and many other people have tried it, with no other effect than soothing the irritation of the membrane to which the spray was applied. The tickling sensation soon ceases, and the mucus is raised with but little effort. In fact, it relieves all the unpleasant symptoms and stops the progress of the catarrh. I believe that it is an absolute cure for all inflammations of the mucus membranes of the nose, throat and lungs, and that it produces the desired effect immediately by contact with the affected part.—*Boston Journal of Medicine.*

